# Bactericidal and Anti-Inflammatory Effects of *Ashitaba*-Extract Ameliorate the Gingivitis and Halitosis in Dogs with *Porphyromonas gulae*-Infected Periodontal Disease

**DOI:** 10.3390/vetsci12100981

**Published:** 2025-10-13

**Authors:** Takayoshi Miyamoto, So Shirahata, Mariko Komuro, Mao Kaneki, Chiharu Ohira, Tomoki Fukuyama

**Affiliations:** 1 Laboratory of Veterinary Pharmacology, School of Veterinary Medicine, Azabu University, 1-17-71, Fuchinobe, Chuo-ku, Sagamihara-shi 252-5201, Kanagawa, Japanda2301@azabu-u.ac.jp (M.K.);; 2 Primo Animal Hospital Kobuchi, JPR Corporation, 4-11-45, Higashi-Fuchinobe, Chuo-ku, Sagamihara-shi 252-0203, Kanagawa, Japan; 3 Center for Human and Animal Symbiosis Science, Azabu University, 1-17-71 Fuchinobe, Chuo-ku, Sagamihara-shi 252-5201, Kanagawa, Japan

**Keywords:** *Ashitaba*-extract, periodontal disease, *Porphyromonas gulae*, dogs, halitosis, gingivitis

## Abstract

**Simple Summary:**

Periodontal disease (PD) is a prevalent oral condition in dogs, often linked to infection by *Porphyromonas gulae* (*P. gulae*). This study examined the antimicrobial, anti-halitosis, and anti-inflammatory effects of *Ashitaba* (*Angelica keiskei*) extract on *P. gulae*-associated PD. In vitro, the extract significantly inhibited bacterial growth, volatile sulfur compound production, and pro-inflammatory cytokine secretion. In vivo, daily oral application of 0.05% *Ashitaba*-extract gel for four weeks improved gingival health, reduced halitosis, and decreased *P. gulae* activity in dogs. These results indicate that *Ashitaba* extract may serve as a potential adjunctive approach for managing canine PD, supporting its further evaluation as a natural bioactive ingredient for maintaining oral health in veterinary applications.

**Abstract:**

*Ashitaba* (*Angelica keiskei*) is a perennial herb native to Japan, traditionally consumed as a health-promoting food and herbal medicine. This study evaluated the antimicrobial, anti-halitosis, and anti-inflammatory effects of *Ashitaba* extract on canine periodontal disease (PD) caused by *Porphyromonas gulae* (*P. gulae*). In vitro, *Ashitaba* extract (0.006–0.1%) significantly inhibited *P. gulae* viability by up to 80% and reduced biofilm formation by approximately 10% at 0.1%. The extract also suppressed the production of volatile sulfur compounds—hydrogen sulfide and methyl mercaptan—by over 80% and 40%, respectively, within 10 min. Furthermore, *Ashitaba* extract markedly decreased *P. gulae*-induced pro-inflammatory cytokine secretion (IL-1β, IL-6, TNF-α) by up to 90% in murine, canine, and human macrophage and gingival cell lines. In vivo, daily oral application of 0.05% *Ashitaba*-extract gel for four weeks, with or without tooth brushing, significantly improved gingivitis scores (by 40–60%), reduced halitosis levels, and decreased *P. gulae* DNA detection and enzymatic activity in dogs with PD. These findings demonstrate that *Ashitaba* extract possesses potent bactericidal, anti-halitosis, and anti-inflammatory properties, supporting its potential as a natural adjunctive therapy for the prevention and management of canine periodontal disease.

## 1. Introduction

With the aging of dogs and cats, periodontal disease (PD) has become one of the most common diseases in veterinary medicine. PD is a prevalent oral health issue in dogs, with studies indicating that approximately 80% of dogs over the age of 3 years are affected [1,2,3]. In Japan, 78.6% of 5-year-old dogs are affected [4]. It is an inflammatory disease caused by bacterial plaques adhering to periodontal tissue and can range from early gingivitis to advanced periodontitis [5]. Once PD progresses to severe periodontitis, damaged gingival tissue and bone lysis never recover. Furthermore, several studies have shown that PD is associated with systemic diseases, such as renal, cardiac, and liver diseases [6,7]. Therefore, preventive dentistry is becoming increasingly important not only for maintaining good oral health but also for the prevention of lifestyle-related diseases.

Previous studies by our group have investigated the preventive and therapeutic effects of catechins and folic acid on canine PD [8,9]. While these compounds demonstrated certain benefits, their mechanisms and efficacy differed, highlighting the need for alternative agents with complementary properties. *Ashitaba* (*Angelica keiskei*), a perennial herb native to Japan, was selected as a promising candidate due to its unique phytochemical profile, which is rich in chalcones, flavanones, and coumarins, known for their antimicrobial, antioxidant, and anti-inflammatory activities. Among these, chalcones such as xanthoangelol and 4-hydroxyderricin are primarily responsible for inhibiting bacterial growth and biofilm formation and have been reported to suppress pro-inflammatory cytokines, primarily via MAPK/p38 activation. NF-κB modulation is suggested based on prior studies. Other constituents, including flavonoids and coumarins, further enhance antioxidant and anti-inflammatory responses, making Ashitaba extract potentially effective against both bacterial virulence and periodontal inflammation [10,11,12,13,14]. Although Ashitaba is widely consumed as a dietary supplement and herbal medicine in Asian countries [15], its biological activity and safety in companion animals remain unclear. Therefore, this study aimed to evaluate the antimicrobial, anti-halitosis, and anti-inflammatory effects of *Ashitaba* extract on canine PD caused by *Porphyromonas gulae (P. gulae)* through in vitro and in vivo analyses.

*P. gulae* has been recognized as a predominant pathogenic bacterium associated with the initiation and progression of periodontitis in dogs [16,17]. Unless PD is properly managed, morbidity and symptoms progress with age, eventually leading to tooth loss. Moreover, recent epidemiological studies indicated that the rate of *P. gulae* infection is significantly correlated with PD stages and the number of residual teeth in dogs with PD. Therefore, in this study, we evaluated the efficacy of *Ashitaba* extract in targeting *P. gulae* in vitro and in vivo.

PD is characterized by plaque accumulation, severe halitosis, and gingival inflammation, particularly during its early stages [18]. Halitosis in companion animals represents a significant concern, as it can negatively affect the emotional bond between pets and their owners [19]. Consequently, improving halitosis through effective PD management can enhance both animal welfare and owner satisfaction. Accordingly, the present study aimed to investigate the antimicrobial, anti-halitosis, and anti-inflammatory effects of *Ashitaba* extract on *P. gulae*-associated PD in dogs using a combination of in vitro assays and in vivo clinical evaluations.

## 2. Materials and Methods

### 2.1. Bactericidal Effects of Ashitaba-Extract on P. gulae

The *Ashitaba* extract used in all in vitro experiments was procured from MARUZEN PHARMACEUTICALS CO., LTD. (Hiroshima, Japan). The *P. gulae* strain ATCC 51700 (fimA type A) was obtained from the Japan Collection of Microorganisms (RIKEN BioResource Research Center, Ibaraki, Japan). The bacteria were cultured anaerobically at 37 °C for 72 h on BD BBL™ CDC Anaerobic 5% Sheep Blood Agar (Becton, Dickinson and Company, Franklin Lakes, NJ, USA). Various concentrations of Ashitaba extract (0.06%, 0.13%, 0.25%, 0.5%, and 1%) were co-incubated with *P. gulae* for 30 min, 1 h, or 4 h. The bactericidal activity was then assessed using the Bac-Titer-Glo™ microbial cell viability assay (Promega KK, Tokyo, Japan) with a luminometer (GloMax^®^ Multi Detection System, Promega KK, Tokyo, Japan).

### 2.2. Evaluation of Hydrogen Sulfide and Methyl Mercaptan Generation by P. gulae

Similarly, *P. gulae* cultures prepared for bactericidal evaluation were co-incubated with *Ashitaba* extract (0.025%, 0.05%, and 0.1%) for 10 min, after which, the production of hydrogen sulfide and methyl mercaptan was quantified using a gas chromatography system (OralChroma, Nissha FIS, Inc., Tokyo, Japan).

### 2.3. Ashitaba Extract Suppresses Pro-Inflammatory Cytokines in P. gulae- and LPS-Stimulated Macrophages

The Ca9-22 (CRL-1629 ™, human gingival carcinoma), J774.1 (TIB-67 ™, mouse monocyte-macrophage), and DH82 cell lines (CRL-3590 ™, canine macrophage) were obtained from the American Type Culture Collection (Manassas, VA, USA). J774.1 cells were cultured in Roswell Park Memorial Institute (RPMI) 1640 medium (FUJIFILM Wako Pure Chemical Corporation, Osaka, Japan) supplemented with 10% fetal calf serum (FCS; Sigma-Aldrich Co., LLC., Tokyo, Japan) and penicillin–streptomycin (FUJIFILM Wako Pure Chemical Corporation). Ca9-22 and DH82 cells were cultured in Eagle’s minimum essential medium (EMEM; FUJIFILM Wako Pure Chemical Corporation) supplemented with 10% fetal FCS and penicillin–streptomycin. Both cell lines (1 × 10^4^ cells/100 μL) were exposed to *Ashitaba* extract (0.006%, 0.013%, 0.025%, 0.05%, and 0.1%) and *P. gulae* (optical density (OD) = 1.0) for 24 h. Levels of IL-1β, IL-6, and TNF-α in the supernatant of Ca9-22, J774.1, and DH82 cells were evaluated using an enzyme-linked immunosorbent assay (ELISA; DuoSet ELISA kit, R&D Systems, Minneapolis, MN, USA). The OD was measured using a microplate reader. The cytotoxicity of the *Ashitaba* extract used in this experiment was assessed using the Cytotoxicity LDH Assay Kit-WST (Dojindo Laboratories, Kumamoto, Japan) before conducting the cytokine release assay. No cytotoxicity was observed even at the highest concentration of *Ashitaba* extract (0.1%).

### 2.4. Effects of Ashitaba Extract on P. gulae-Induced p38 Phosphorylation in DH82 Cells

The phosphorylation levels of p38 in DH82 cells were assessed 1 h after treatment with *Ashitaba* extract (0.05% and 0.1%) and exposure to *P. gulae* (1 × 10^4^ CFU/mL) using a Western blot analysis. Total proteins (30 µg) were extracted from the cells with M-PER™ Mammalian Protein Extraction Reagent (Thermo Fisher Scientific, Sagamihara-shi, Kanagawa, Japan), separated by SDS-PAGE, and transferred onto PVDF membranes using the Trans-Blot Turbo Transfer System (Bio-Rad Laboratories, Inc., Tokyo, Japan). Protein detection was performed with primary antibodies against phospho-p38, total p38, and β-actin (Abcam plc., Cambridge, UK), followed by visualization with secondary antibodies and detection using ImmunoStar^®^ Zeta (FUJIFILM Wako Pure Chemical Corporation). Protein bands were quantified with the iBright imaging system (Thermo Fisher Scientific).

### 2.5. Daily Oral Ashitaba-Extract Treatments in Dogs with P. gulae-Positive PD

All experimental protocols were approved by the Animal Care and Use Program of Azabu University (Approval No. 200318-1). All methods complied with the ARRIVE guidelines (https://arriveguidelines.org, accessed on 18 March 2020) and relevant animal welfare regulations. Written informed consent was obtained from all pet owners before participation. A total of 45 dogs (4–16 years old; Table 1) with moderate-to-severe PD and no prior dental care within the preceding month were enrolled. A sample size of 45 was determined through power analysis (α = 0.05, power = 0.8), assuming a 30% expected improvement in gingivitis scores. Dogs were randomly assigned to one of four groups: untreated control (*n* = 5), tooth brushing only (*n* = 10), *Ashitaba*-extract gel (0.05%) only (*n* = 15), and *Ashitaba*-extract gel (0.05%) + tooth brushing (*n* = 15). Randomization was performed using a computer-generated allocation sequence. Clinical assessments were conducted by a blinded veterinarian unaware of treatment assignments to minimize bias. Dental gel and/or tooth brushing were administered once daily for 30 min after the evening meal for four weeks. Plaques and gingivitis were visually evaluated on the most severely affected teeth using a 0–3 scale (0 = normal, 3 = severe) without anesthesia or radiographs. These procedures were omitted to minimize animal stress and ensure owner consent, in accordance with ethical guidelines for non-invasive clinical studies. Although this approach may introduce some degree of observational bias compared with radiographic or anesthetized assessments, all evaluations were performed by the same blinded veterinarian under standardized lighting and positioning conditions to maintain consistency and reduce subjectivity. Halitosis indicators—hydrogen sulfide and methyl mercaptan—were measured in expired air using gas chromatography (Oral Chroma). *P. gulae* detection in oral swabs from the gingival margin and sulcus of the maxillary canine or fourth premolar was performed using a polymerase chain reaction (PCR) assay [8]. Enzymatic activity associated with severe periodontal pathogens (*Treponema denticola*, *P. gingivalis*, *P. gulae*, and *Bacteroides forsythus*) was further assessed using the BANA test (BANAMet LLC, Ann Arbor, MI, USA) [20].

### 2.6. Statistical Analyses

All data are presented as the mean ± standard error of the mean (SEM). For in vitro experiments involving multiple groups, differences were assessed using analysis of variance (ANOVA) followed by Dunnett’s or Šídák’s multiple comparisons test. For clinical studies, two-way ANOVA was applied, followed by Tukey’s multiple comparisons test. Statistical significance was set at a probability level of 5% (*p* < 0.05). All analyses were performed using GraphPad Prism 10 (GraphPad Software, San Diego, CA, USA).

## 3. Results

### 3.1. Ashitaba Extract Inhibits the Viability and Biofilm Formation of P. gulae

To elucidate the antimicrobial effect of *Ashitaba* extract on *P. gulae*, the extract (0.006–0.1%) was incubated with *P. gulae* for 30 min to 4 h, and bacterial viability was evaluated based on ATP content. *Ashitaba* extract significantly reduced *P. gulae* viability in a concentration-dependent manner from 30 min post-treatment. In contrast, no significant inhibition of biofilm formation was observed during this short exposure period; notable suppression of biofilm was only detected after 72 h of treatment at concentrations of 0.05% and 0.1%.

### 3.2. Ashitaba Extract Reduced P. gulae-Derived Hydrogen Sulfide and Methyl Mercaptan

**Figure 1:**
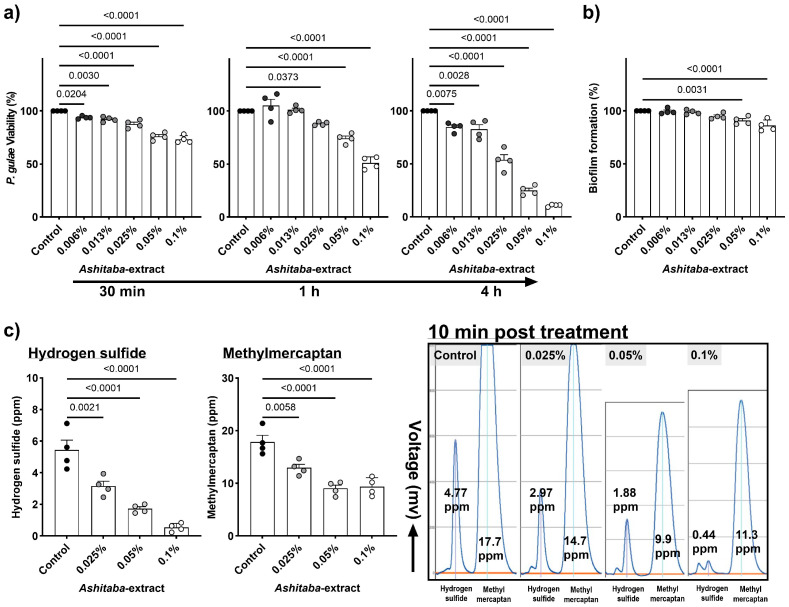
The direct influence of *Ashitaba* extract on *P. gulae* activities. (**a**) Bactericidal effects of *Ashitaba* extract against *P. gulae* at each time point. (**b**) Biofilm formation of *P. gulae* was significantly inhibited following 72 h of *Ashitaba*-extract treatment. (**c**) Hydrogen sulfide and methyl mercaptan production by *P. gulae* following 10 min co-incubation with *Ashitaba* extract. Representative images of gas chromatography are also indicated. Data are presented as the mean (% or ppb) ± 1 SEM, with *n* = 4 per group. Black dots represent individual data points for the control group, and gray to white dots indicate data points for the *Ashitaba*-extract–treated group. Statistical significance (*p* < 0.05) was determined using Dunnett’s multiple comparison test versus the control (0%) group. *P. gulae*, *Porphiromonas gulae*.

Halitosis is not directly associated with PD pathogenesis. However, it negatively influences the quality of life and social aspects. Hydrogen sulfide and methyl mercaptan, which are the key elements of halitosis produced by *P. gulae*, were significantly reduced by the *Ashitaba*-extract treatment (0.025%, 0.05%, and 0.1%) in a dose-dependent manner, although in a very short period of exposure (10 min) (Figure 1c).

### 3.3. P. gulae-Stimulated Cytokine Production Was Inhibited by Ashitaba-Extract

**Figure 2:**
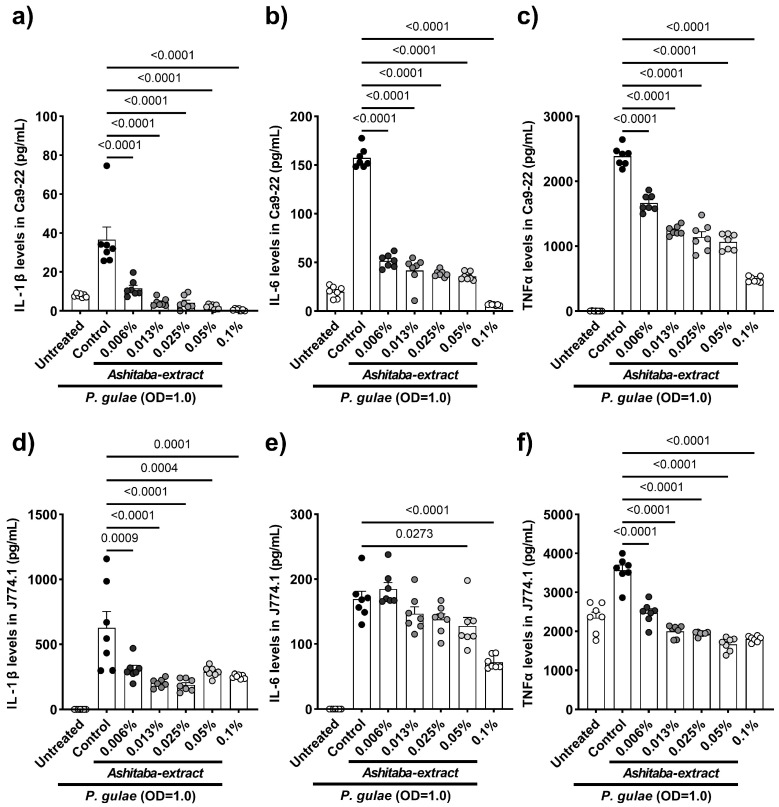
Inhibitory effects of *Ashitaba* extract on the production of pro-inflammatory cytokines by human gingival cell line (Ca9-22) and murine macrophage cell line (J774.1). (**a**) IL-1β, (**b**) IL-6, and (**c**) TNF-α secretion by the *P. gulae*-infected Ca9-22. (**d**) IL-1β, (**e**) IL-6, and (**f**) TNF-α secretion by the *P. gulae*-infected J774.1. Each result is the mean (pg/mL) ± 1 SEM. *n* = 7 per group. Black dots represent individual data points for the control group, and gray to white dots indicate data points for the *Ashitaba*-extract–treated group. *p* < 0.05 (Dunnett’s multiple comparison test) vs. *P. gulae*-infected control group. IL, interleukin; TNF, tumor necrosis factor.

**Figure 3:**
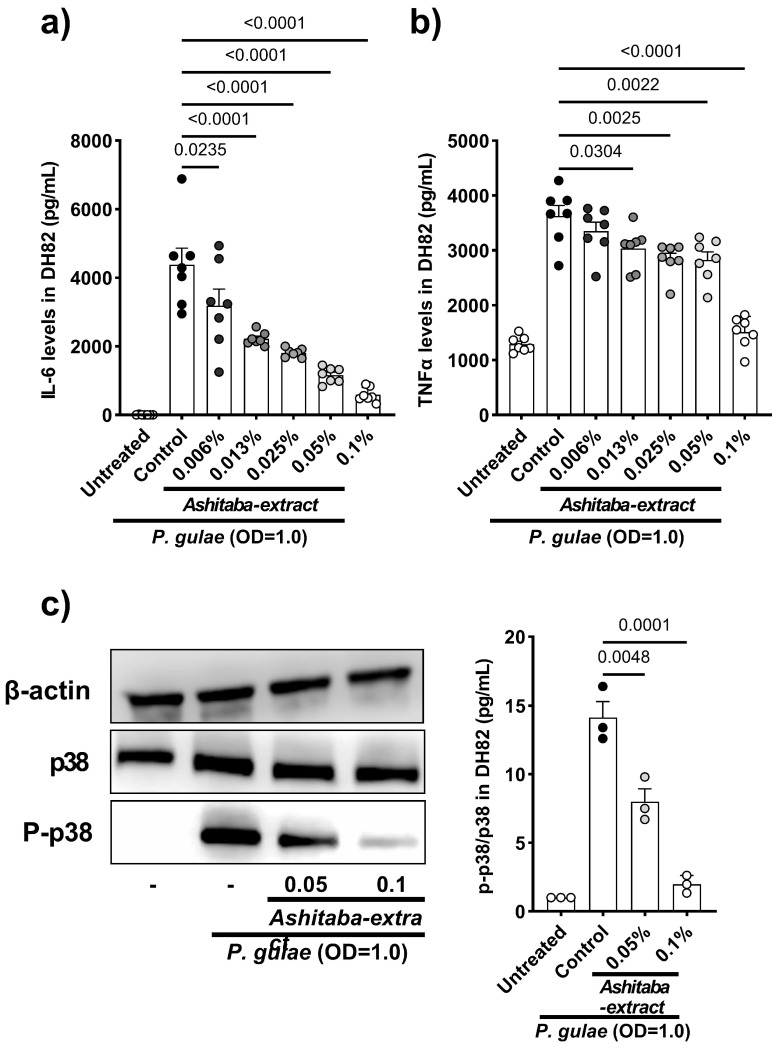
Inhibitory effects of *Ashitaba* extract on the production of pro-inflammatory cytokines and phosphorylation of p38 induced by *P. gulae* by canine macrophage cell line (DH82). (**a**) IL-6 and (**b**) TNF-α secretion by the *P. gulae*-infected canine macrophage. (**c**) Representative images of Western blot analysis. The grouping of gels/blots cropped from different parts of the same gel, and exposures were made explicit. *Ashitaba*-extract treatment significantly reduced the phosphorylation of p38 induced by *P. gulae* infection in a dose-dependent manner. Each result is the mean ± standard error of the mean (SEM). N = 4 (Western blot) or 7 (ELISA) per group. Black dots represent individual data points for the control group, and gray to white dots indicate data points for the *Ashitaba*-extract–treated group. *p* < 0.05 (Dunnett’s multiple comparison test) vs. control group. (Original image is in Supplementary Materials Figures S1–S3).

Infection of gingival tissue by periodontal pathogens or their endotoxin (lipopolysaccharide) and immunocytes, such as macrophages, induces proinflammatory cytokine production and leads to gingivitis and bone lysis. In the next step, whether the direct effects of *Ashitaba* extract can suppress inflammatory cytokines (IL-1β, IL-6, TNFα) production by gingival (Ca9-22) and macrophage (J774.1) cell lines are shown. Our results indicated that 24 h co-incubation with *Ashitaba*-extract (0.006–0.1%) significantly suppressed *P. gulae*-induced cytokine production in a dose-dependent manner (Figure 2a–f). A similar investigation was performed using the canine macrophage cell line, DH82, to translate data from murine and human cell lines into canine cells. Co-treatment with *Ashitaba* extract for 24 h also significantly reduced *P. gulae*-induced production of IL-6 and TNF-α (Figure 3a,b). The secretion of pro-inflammatory cytokines is regulated by the mitogen-activated protein kinase pathway that includes p38 and NFκB. The effects of *Ashitaba* extract on *P. gulae*-induced phosphorylation of p38 were assessed in DH82 cells. *Ashitaba*-extract treatment at concentrations of 0.05% and 0.1% significantly inhibited *P. gulae*-induced phosphorylation of p38 in a dose-dependent manner (Figure 3c, Figures S1–S3).

### 3.4. Ashitaba-Extract Gel (0.05%) Alleviates PD Symptoms in P. gulae-Infected Dogs, With or Without Brushing

**Figure 4:**
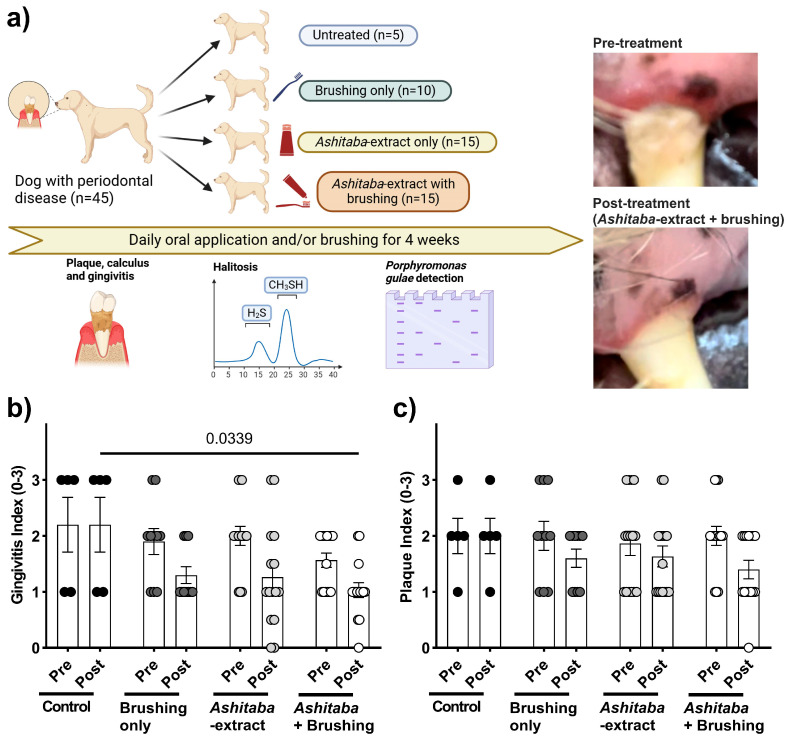
Changes in periodontal conditions before and after *Ashitaba*-extract (0.05%) gel treatment and/or tooth brushing in dogs infected with *P. gulae*. (**a**) Experimental schedule and groups of this study. (**b**) Gingivitis was significantly ameliorated by the *Ashitaba*-extract treatment and/or tooth brushing compared to the pre-treatment value. (**c**) The plaque index was significantly ameliorated by the *Ashitaba*-extract treatment and tooth brushing compared to the pre-treatment value. Each result is mean ± 1 SEM. Black, dark gray, gray and white dots represent individual data points for the control, brushing only, *Ashitaba*-extract, and *Ashitaba*+Brushing groups, respectively. *p* < 0.05 (Šídák’s multiple comparisons test).

**Figure 5:**
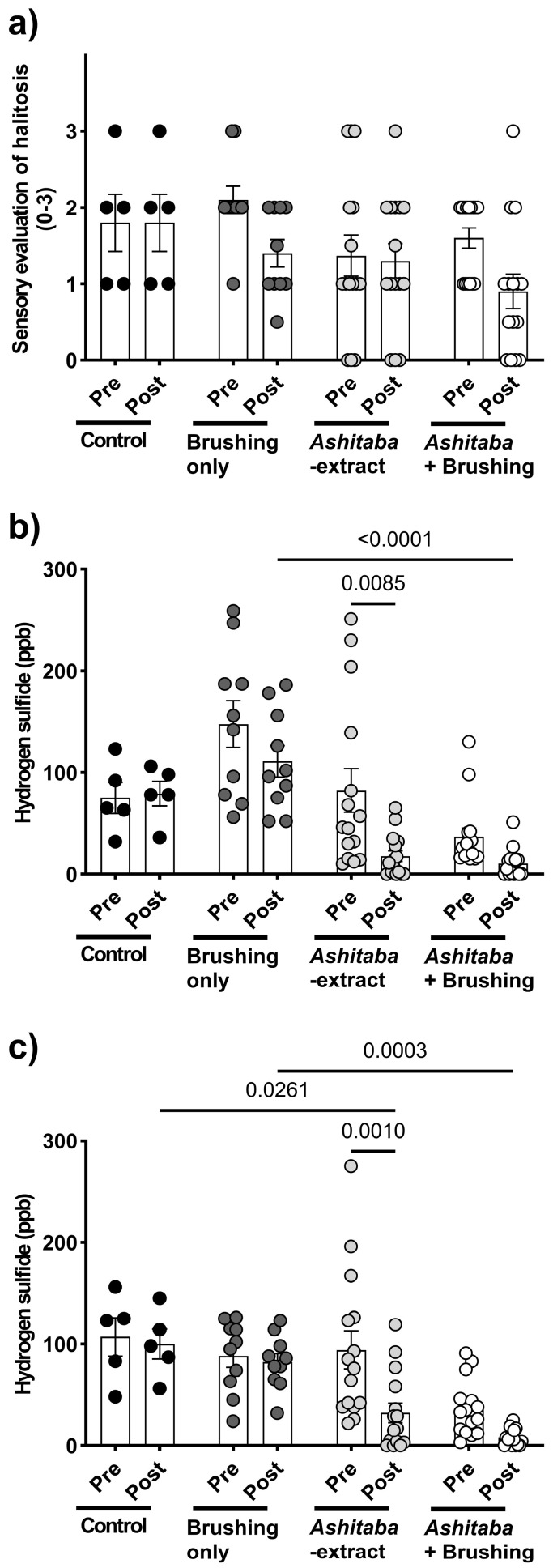
Changes in halitosis before and after *Ashitaba*-extract (0.05%) gel treatment and/or tooth brushing in dogs infected with *P. gulae*. (**a**) Pet owners’ sensory evaluation of halitosis showed significant improvement following *Ashitaba*-extract treatment and tooth brushing compared to pre-treatment and vehicle control values in dogs. (**b**) Hydrogen sulfide (ppb) and (**c**) methyl mercaptan (ppb) levels in dog breath were significantly reduced by *Ashitaba*-extract treatment compared to pre-treatment and vehicle control values in dogs. Each result is mean ± 1 SEM. Black, dark gray, gray and white dots represent individual data points for the control, brushing only, *Ashitaba*-extract, and *Ashitaba*+Brushing groups, respectively. *p* < 0.05 (Šídák’s multiple comparisons test).

**Figure 6:**
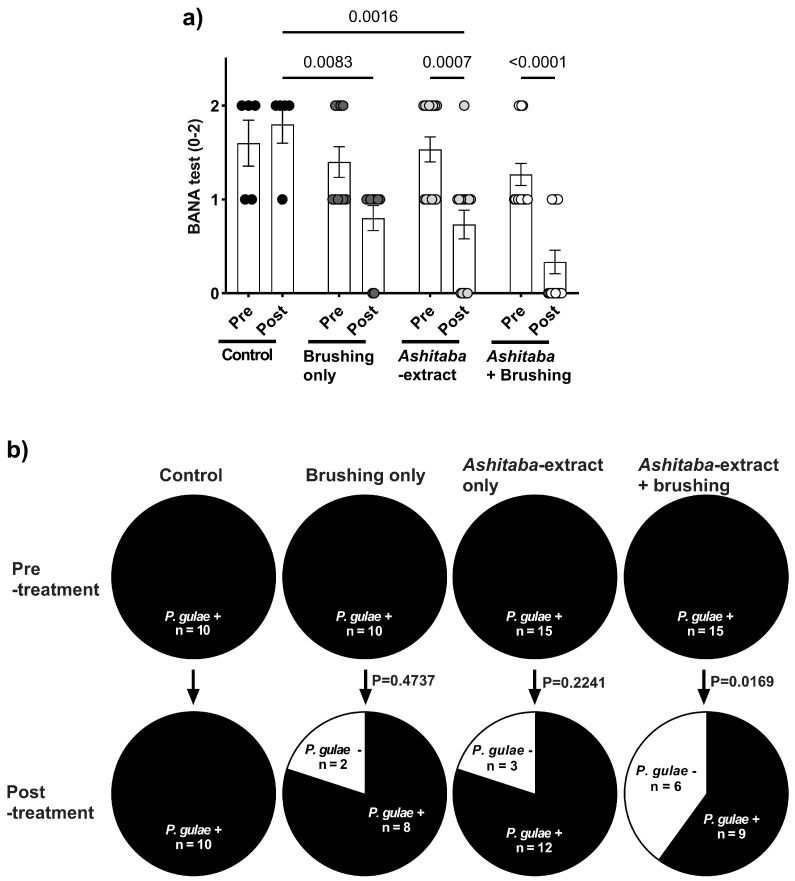
Impact on *P. gulae* activity and DNA detection before and after *Ashitaba*-extract (0.05%) gel treatment and/or tooth brushing in dogs infected with *P. gulae*. (**a**) BANA levels were significantly suppressed by the *Ashitaba*-extract treatment and/or tooth brushing compared to the pre-treatment levels and that in the vehicle control groups. (**b**) DNA detection of *P. gulae* was significantly suppressed by the *Ashitaba*-extract treatment and tooth brushing compared to the pre-treatment levels. Each result is mean ± 1 SEM. Black, dark gray, gray and white dots represent individual data points for the control, brushing only, *Ashitaba*-extract, and *Ashitaba*+Brushing groups, respectively. *p* < 0.05 (Šídák’s multiple comparisons test). BANA, N-benzoyl-DL-arginine-2-naphthylamide; PCR, polymerase chain reaction.

To confirm the in vitro antimicrobial, anti-halitosis, and anti-inflammatory effects of *Ashitaba* extract, a 4-week clinical study was conducted in dogs with *P. gulae* infection (Figure 4a). Daily treatment with 0.05% *Ashitaba*-extract gel, combined with tooth brushing, significantly reduced the gingivitis index by 40–60% compared to untreated controls (Figure 4b), whereas the plaque index was not significantly affected (Figure 4c). Halitosis evaluation revealed that pet-owner sensory scores were not significantly altered by *Ashitaba*-extract treatment alone. However, volatile sulfur compound levels—including hydrogen sulfide and methyl mercaptan—were significantly decreased by both *Ashitaba*-extract treatments with and without brushing (Figure 5b,c). Bactericidal effects were confirmed via BANA-degrading enzyme activity and *P. gulae* DNA detection. *Ashitaba* extract combined with brushing significantly reduced both BANA activity and *P. gulae* DNA detection compared to pre-treatment and control groups, whereas *Ashitaba* extract alone modestly decreased BANA activity with a minimal effect on DNA detection (Figure 6a,b). The observed reductions in gingivitis (40–60%) and volatile sulfur compounds (VSCs) in expired breath are consistent with or exceed the improvements reported in recent veterinary studies. For example, a study by Gawor, et al. [21] demonstrated that a water additive containing pomegranate significantly decreased plaque and calculus accumulation and improved gingival health in dogs after 30 days of use. Additionally, a clinical trial by Sordillo, et al. [22] reported a 27% reduction in VSCs in dogs treated with a novel postbiotic, highlighting the potential for non-mechanical interventions to manage halitosis. These findings suggest that *Ashitaba*-extract treatment provides clinically meaningful improvements in canine periodontal health and can serve as an effective adjunct to standard interventions.

## 4. Discussion

PD is an inflammatory disease characterized by periodontitis and gingivitis caused by dental plaque, a bacterial mass that forms in the gingival sulcus between the teeth and gums [5]. In veterinary medicine, PD is prevalent in dogs and cats, and its progression can lead to tooth loss and the development of systemic diseases [2]. Periodontal disease is known to cause halitosis and is an important factor in relationships with owners [18,19]. If the condition advances to the stage where the teeth become loose, extraction is the only viable treatment. Therefore, intervention and routine dental care are advised when symptoms are still mild [23]. In this study, we examined the effects of *Ashitaba* extract on periodontal disease and halitosis in dogs.

*Ashitaba* extract is an antimicrobial substance reported to be effective mainly against Gram-positive bacteria. Previous studies have shown that its antibacterial activity is primarily mediated by chalcones, such as xanthoangelol and 4-hydroxyderricin, which disrupt bacterial membranes, inhibit biofilm formation, and suppress bacterial metabolism, thereby reducing the growth and virulence of pathogens such as *P. gulae* [12,14,24]. Initially, *Ashitaba* extract at concentrations of 0.006–0.1% was tested for its inhibitory effect on the viability of *P. gulae*, a major contributor to periodontitis in dogs and commonly used as an in vitro indicator for evaluating anti-periodontitis effects in veterinary research [25]. Significant antimicrobial activity against *P. gulae* was observed from 30 min to 4 h after *Ashitaba*-extract treatment. In our previous study, catechin treatment showed antimicrobial properties comparable to *Ashitaba* extract [8]. However, significant effects were observed 4 h after catechin treatment, whereas *Ashitaba* extract indicated earlier effects 30 min after treatment. In addition, catechin treatment only showed a significant inhibition of 0.022%, whereas *Ashitaba*-extract treatment was significantly effective at concentrations higher than 0.006%. Regarding microbial properties, *Ashitaba* extract provides better benefits than natural agents such as catechin. The antimicrobial properties of *Ashitaba* extract were also demonstrated in a clinical trial using a BANA-degrading active enzyme and *P. gulae* DNA detection. We found that periodontal bacterial activity was reduced after one month of treatment with either dental gel alone or with a combination of dental gel and brushing. The number of detected *P. gulae* DNA was reduced in the dental gel and brushing combination treatment, suggesting that the additional brushing treatment decreased the detection rate of *P. gulae*. Tooth brushing has been reported to inhibit the growth of oral bacteria [26]. However, brushing alone was not sufficient to affect periodontal bacterial activity and detection significantly. The combination of *Ashitaba*-extract treatment and brushing is reasonable for pet owners as a home-care application.

On the other hand, our findings demonstrated that the inhibitory effect on biofilm formation by *P. gulae* was only detected at more than 0.05% of *Ashitaba* extract after 72 h of treatment. Similar results were obtained in a clinical study in which *Ashitaba* extract did not alter the plaque index, whereas BANA-degrading active enzymes were significantly suppressed by *Ashitaba*-extract treatment alone. This should be a limitation of supplemental agents, including *Ashitaba*-extract treatment. Our previous study focused on the effects of catechin on canine and feline PD, and similar limitations in the inhibitory effects on biofilm formation were observed [8]. However, in this study, the combination of *Ashitaba*-extract treatment and tooth brushing affected the plaque index significantly. Based on our results, this is an advantage of *Ashitaba*-extract treatment, as the dental plaque of moderate-to-severe PD is not removable only by brushing. While mechanical applications like brushing and scaling are the first options for removing dental plaque and calculus, frequent scaling is unavailable because general anesthesia is mandatory for dogs and cats. The combination of *Ashitaba*-extract treatment and tooth brushing by the pet owner would be a reasonable application for preventing and removing plaque.

Halitosis is strongly associated with periodontitis and results from volatile sulfur compounds (VSCs), including hydrogen sulfide and methyl mercaptan, which are produced by periodontal bacteria in the oral cavity [27]. In this study, treatment with 0.025–0.1% *Ashitaba* extract significantly suppressed the production of hydrogen sulfide and methyl mercaptan in only 10 min in vitro. The anti-halitosis effect of *Ashitaba* extract was also confirmed in a clinical study. Interestingly, significant effects of *Ashitaba* extract on the levels of hydrogen sulfide and methyl mercaptan were observed only in the *Ashitaba*-extract treatment without the tooth-brushing group, compared to the pre-treatment and vehicle control values. This may be because the pre-treatment levels of hydrogen sulfide and methyl mercaptan in *Ashitaba* extract with the tooth-brushing group were low. However, our findings suggest that *Ashitaba* extract may provide beneficial effects against *P. gulae*-associated PD in dogs, although further studies are needed to confirm its long-term efficacy.

Bacterial components and compounds generally induce the inflammatory responses to PD in macrophages and gingival cells [28,29]. In our study, the effect of *Ashitaba* extract on *P. gulae*-related inflammatory responses was examined in vitro. The human gingival carcinoma cell line Ca9-22 and the murine macrophage cell line J774.1 have been widely used to evaluate inflammatory responses in PD in vitro [30,31,32]. In this study, the canine macrophage cell line DH82 was also used to translate human and murine data into dogs, as the demonstration of efficacy in dogs was the final goal of this study. Our results showed that the co-incubation of *Ashitaba* extract with *P. gulae* significantly decreased the production of IL-1β, IL-6, and TNF-α in Ca9-22, J774.1, and DH82 cell lines. In general, the antimicrobial property of *Ashitaba* extract on *P. gulae* inhibits the pro-inflammatory cytokine production by gingival and macrophage cell lines. However, a previous study reported that the n-hexane fraction of *Ashitaba* exerted anti-inflammatory effects by suppressing mitogen-activated protein kinases (MAPKs) [33]. Thus, we examined the effect of *Ashitaba*-extract on the phosphorylation of p38, a member of the mitogen-activated protein kinase family, and found significant inhibition 1 h after treatment with 0.05% and 0.1% *Ashitaba*-extract. The antimicrobial effects of *Ashitaba*-extract should be included in this anti-inflammatory response. However, the phosphorylation of p38 in the 0.1% treatment group was almost the same as that in the untreated group. The anti-inflammatory responses of *Ashitaba* extract might consist of antimicrobial properties against periodontal bacteria and anti-inflammatory properties through suppressing MAPKs. Anti-inflammatory responses to *Ashitaba*-extract treatment were confirmed using the gingivitis index of the clinical study. *Ashitaba*-extract treatment with or without tooth-brushing significantly ameliorated the gingival index compared to the pre-treatment and vehicle control values. This is the biggest advantage of *Ashitaba* extract compared to other natural components. In our previous clinical study, catechins, persimmon tannin, and folic acid did not suppress gingivitis [8,9]. However, the formulation of the oral care items (dental gel in this case) has a big impact on the results.

To the best of our knowledge, this is the first study to demonstrate that *Ashitaba* extract is effective against the pathogenesis of PD in dogs. We observed significant antimicrobial, anti-halitosis, and anti-inflammatory effects against *P. gulae* in vitro, which were corroborated in a clinical study involving dogs with *P. gulae*-infected PD. Four weeks of daily intraoral treatment with 0.05% *Ashitaba*-extract gel significantly reduced gingivitis, halitosis, and periodontal bacterial activity. While mechanical cleaning, such as tooth brushing or professional scaling, physically removes plaque and calculus, *Ashitaba* extract directly targets *P. gulae*, inhibits biofilm formation, suppresses pro-inflammatory cytokine release via MAPK/p38 activation (with NF-κB modulation suggested by previous reports), and reduces volatile sulfur compounds, contributing to improved oral comfort. Combining *Ashitaba* extract with mechanical cleaning may enhance the efficacy of standard therapies and help maintain gingival health. However, the extract alone showed limited effects on plaque formation, indicating that mechanical removal remains essential. This study has several other limitations. The sample size was relatively small, and clinical assessments were conducted without anesthesia or radiographs, which may introduce observational bias. The trial duration was limited to four weeks, preventing evaluation of long-term efficacy and safety. Although *Ashitaba* extract exhibited concentration- and time-dependent antimicrobial and anti-biofilm effects in vitro, the 0.05% gel applied once daily may not fully maintain the effective concentration or exposure time in the oral cavity, potentially limiting biofilm inhibition. Salivary dilution and mechanical removal of plaque could reduce local activity. Furthermore, the specific bioactive components within *Ashitaba* responsible for these effects remain unclear, representing an important area for future mechanistic studies. Future research should also explore formulations that enhance retention or provide controlled release, assess optimal dosing frequency, and evaluate long-term outcomes in larger, controlled cohorts to better establish clinical efficacy and safety.

## 5. Conclusions

This study provides preliminary evidence that *Ashitaba* extract exhibits antimicrobial, anti-halitosis, and anti-inflammatory effects against *P*. *gulae*, a key pathogen in canine PD. In vitro, *Ashitaba* extract (0.006–0.1%) reduced *P. gulae* viability by up to 80% and inhibited biofilm formation by approximately 10% at 0.1%. Hydrogen sulfide and methyl mercaptan production were suppressed by over 80% and 40%, respectively, within 10 min. The extract also decreased IL-1β, IL-6, and TNF-α secretion by up to 90% in human, murine, and canine cell lines and reduced p38 MAPK phosphorylation, suggesting modulation of the inflammatory pathways. In vivo, daily application of 0.05% *Ashitaba*-extract gel for four weeks reduced gingivitis scores by 40–60%, lowered volatile sulfur compounds in breath, and decreased *P. gulae* DNA detection and BANA enzyme activity, particularly when combined with tooth brushing. While these results are promising, the sample size was limited, plaque reduction required mechanical intervention, and long-term safety and efficacy were not assessed. Future studies with larger controlled cohorts, extended follow-up, and mechanistic analyses are needed to validate these findings and clarify the potential role of *Ashitaba* extract in canine oral health management.

## Figures and Tables

**Table 1:** Dog breeds included in the clinical study.

Group	Breeds	Age	Sex
Untreated group (*n* = 5)	Toy Poodle	12 years	Male (castration)
	Shiba	7 years	Female (spay)
	Miniature Dachshund	8 years	Female (spay)
	Miniature Dachshund	<10 years	Female (spay)
	Toy Poodle	6 years	Male (castration)
Brushing-only group (*n* = 10)	Toy Poodle	8 years	Male (castration)
	Toy Poodle	6 years	Female (spay)
	Toy Poodle	14 years	Female (spay)
	Miniature Dachshund	unknown	Female (spay)
	Chihuahua	5 years	Female (spay)
	Miniature Dachshund	7 years	Female (spay)
	Chihuahua	12 years	Male (castration)
	Chihuahua	8 years	Female (spay)
	Beagle	10 years	Female (spay)
	Italian Greyhound	6 years	Male (castration)
*Ashitaba*-extract group (*n* = 15)	Yorkshire terrier	10 years	Female (spay)
	Toy Poodle	5 years	Male (castration)
	Miniature Dachshund	6 years	Female (spay)
	Maltese × Toy Poodle	5 years	Male (castration)
	Miniature Dachshund	unknown	Female (spay)
	Miniature Dachshund	5 years	Male (castration)
	Toy Poodle	16 years	Male (castration)
	Miniature Dachshund	11 years	Female (spay)
	Miniature Dachshund	<10 years	Female (spay)
	Toy Poodle	4 years	Female (spay)
	Chihuahua	6 years	Male (castration)
	Pomeranian	5 years	Female (spay)
	Italian Greyhound	9 years	Female (spay)
	Pomeranian	11 years	Male (castration)
*Ashitaba*-extract + Brushing group (*n* = 15)	Yorkshire terrier	11 years	Female (spay)
	Miniature Dachshund	9 years	Female (spay)
	Miniature Dachshund	8 years	Female (spay)
	Toy Poodle	5 years	Male (castration)
	Toy Poodle	6 years	Female (spay)
	Chihuahua	13 years	Female (spay)
	Toy Poodle	unknown	Male (castration)
	Chihuahua	11 years	Female (spay)
	Chihuahua	<10 years	Female (spay)
	Toy Poodle	4 years	Female (spay)
	Jack Russell	unknown	Male (castration)
	Pomeranian	5 years	Female (spay)
	Italian Greyhound	8 years	Female (spay)
	Pomeranian	7 years	Male (castration)
	Jack Russell	8 years	Male (castration)

## Data Availability

The raw data supporting the conclusions of this article will be made available by the authors on request.

## Supplementary Materials

The following supporting information can be downloaded at: https://www.mdpi.com/article/10.3390/vetsci12100981/s1, Figure S1, Original Western blot images related to Figure 3c (β-actin). Figure S2, Original Western blot images related to Figure 3c (p38). Figure S3, Original Western blot images related to Figure 3c (p-p38).

## Abbreviations

The following abbreviations are used in this manuscript:

PD	Periodontal disease
*P. gulae*	*Porphyromonas gulae*
RPMI	Roswell Park Memorial Institute
ELISA	Enzyme-linked immunosorbent assay
BANA	N-benzoyl-DL-arginine-naphthylamide
SEM	Standard error of the mean
ANOVA	Analysis of variance
VSC	Volatile sulfide compounds
MAPKs	Mitogen-activated protein kinases
