# Supplementary material for: Bactericidal and Anti-Inflammatory Effects of Ashitaba-Extract Ameliorate the Gingivitis and Halitosis in Dogs with Porphyromonas gulae-Infected Periodontal Disease
Source: Vet Sci. 2025 Oct 13;12(10):981. doi: 10.3390/vetsci12100981 (PMC12567811; doi:10.3390/vetsci12100981)

Figure S1 Original Western blot images related to Figure 3c (β -actin).

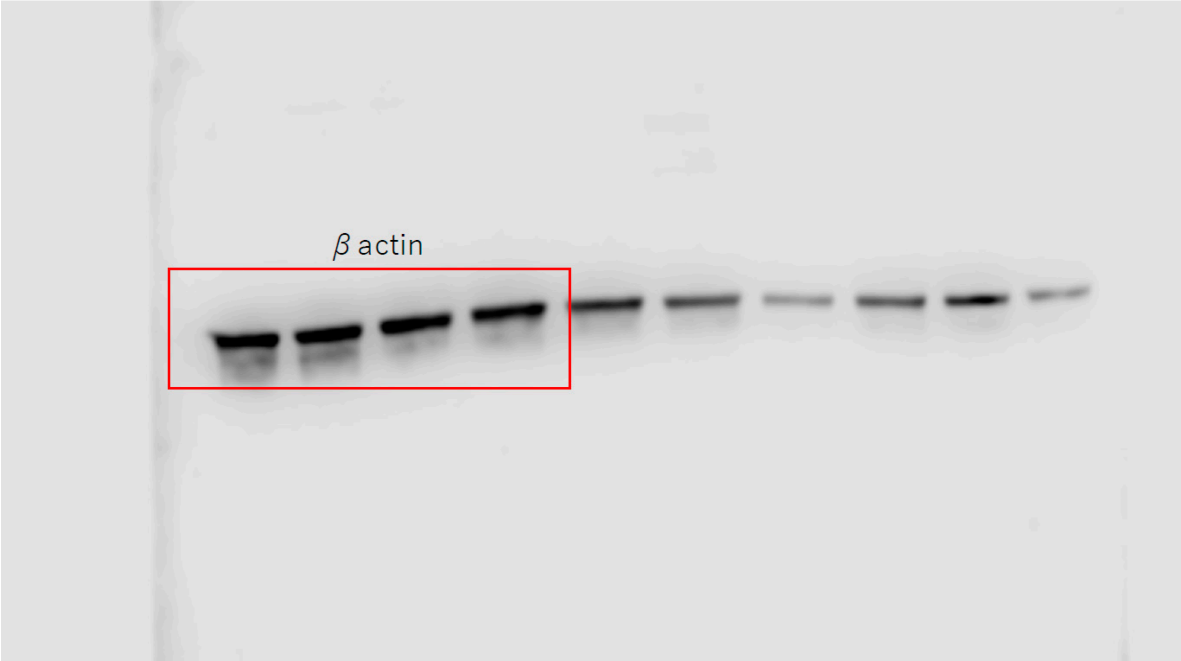

Figure S2 Original Western blot images related to Figure 3c (p38).

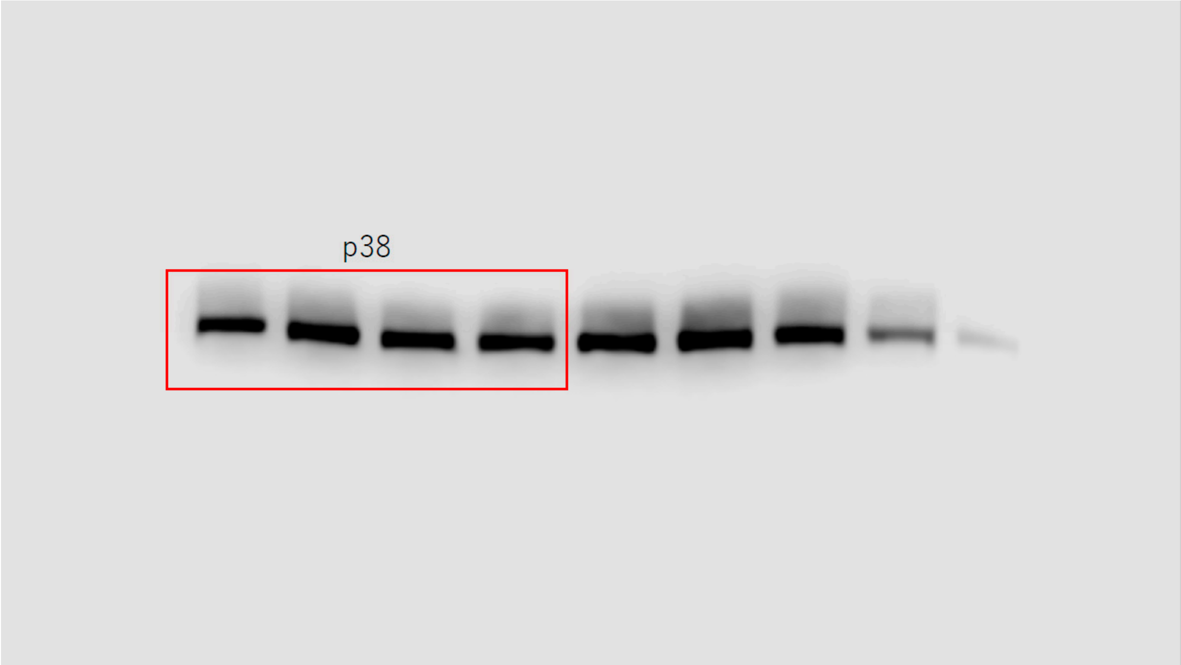

Figure S3 Original Western blot images related to Figure 3c (p-p38).

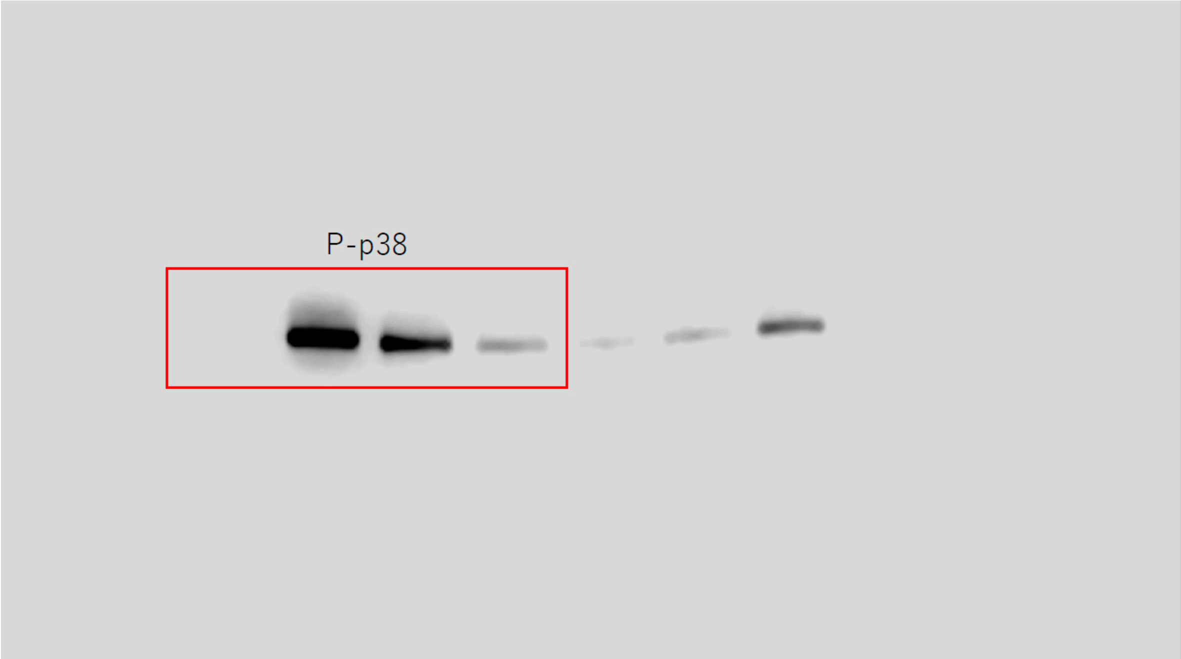

Supplement: Supplementary file 1 [file vetsci-12-00981-s001.zip › vetsci-3907656-supplementary.pdf]
